# Diet in Gout

**Published:** 1890-06-14

**Authors:** 


### DIET IN GOUT.

It is usual to prescribe for gouty patients great moderation in the consumption of meat, under the impression that the formation of uric acid will be thus restricted. But the more easily any article of food of the class of albumens is assimilated the greater is the relative production of urea, and the less that of uric acid, the latter being the result of imperfect metabolism, whether dependent on the ingestion of an undue amount of nitrogenous food stuffs, defective assimilation, or excessive tissue waste, as in febrile states. Dr. Schultze, from experiments on himself, as well as on dogs, finds that the more a diet approaches to a purely animal one the greater the proportion of nitrogen eliminated as urea, and the less, relatively, as uric acid. This proportion, indicative of more complete metabolism, is favoured by alkaline drinks, but opposed by the use of alcohol. As one's aim in the treatment of gout is to minimize the formation of the less soluble and less easily eliminated urates, the value of alkalies and the importance of prohibiting alcoholic drinks is evident, while, the vegetable albumens being far less digestible than those of animal origin, it would appear that the latter, in amounts, of course, adapted to the actual nitrogenous demands of the organism, are to be preferred.

